# Assessment of the utility of platelet indices to diagnose clinical benign prostatic hyperplasia in dogs

**DOI:** 10.3389/fvets.2022.1031292

**Published:** 2022-12-08

**Authors:** Hediyeh Hosseinpour, Mahmood Ahmadi-hamedani, Majid Masoudifard, Darush Shirani, Reza Narenj Sani

**Affiliations:** ^1^Department of Clinical Sciences, Faculty of Veterinary Medicine, Semnan University, Semnan, Iran; ^2^Department of Surgery and Radiology, Faculty of Veterinary Medicine, University of Tehran, Tehran, Iran; ^3^Department of Internal Medicine, Faculty of Veterinary Medicine, University of Tehran, Tehran, Iran

**Keywords:** area under curve, dogs, BPH, platelet indices, relative prostatic size

## Abstract

**Introduction:**

Platelet indices changes in severely ill people and in dogs with inflammation are compatible findings. This study aimed to compare platelet indices between dogs with clinical benign prostatic hyperplasia (BPH) and healthy controls. Additionally, to determine whether there is a correlation between the relative prostatic size (*S*_rel_) and the platelet indices in BPH dogs.

**Methods:**

Thirty-five adult intact male dogs of different breeds were allocated to the experimental groups: dogs with clinical BPH (groups A; *n* = 24; median age of 6 years; the median weight of 8.50 kg) and healthy dogs (group B; *n* = 11; median age 5.50 years; the median weight of 7.00 kg) based on physical examination, clinical signs, and *S*_rel_ detected by ultrasonographic findings. The individual prostatic volume (IPV) was divided by the expected prostatic volume (EPV) to determine the relative prostatic size in dogs over 4 years old. Platelet indices were compared between the two groups, and a correlation between *S*_rel_ and these indices was calculated.

**Results:**

The median *S*_rel_ of dogs in group A was significantly higher (*P* = 0.001), and the mean plateletcrit (PCT) was significantly lower (*P* = 0.003) compared with those in group B. *S*_rel_ showed a significant negative correlation with PLT and PCT (*r* = −0.388; *P* = 0.02 and *r* = −0.402; *P* = 0.01). Receiver operating characteristic (ROC) analysis showed PLT and PCT thresholds for estimating *S*_rel_ > 1 with 75% and 87.5% sensitivity and 71.82 and 63.64% specificity.

**Discussion:**

The findings of this study support the use of platelet indices like PLT and PCT to detect clinical BPH in dogs. However, more research is needed to confirm their utility in conjunction with other previously described diagnostic factors.

## Introduction

Benign prostatic hyperplasia (BPH) is the most common prostate disease in intact male dogs (1–3). BPH is an age-related spontaneous disease that many pet owners overlook (4, 5). Although the pathogenesis of BPH is unknown, androgen derivatives, androgen-to-estrogen ratio, and inflammation caused by chemokine released from the prostatic stroma all play a role in its development (6). A study of intact male dogs older than 5 and 9 years revealed that BPH affected 80 and 95% of them, respectively (7). Periodic bloody discharge, haematuria, haematospermia, dyschezia, strangury, incontinence, constipation, tenesmus, and stilted gait are the most common clinical signs of BPH (8). History, clinical signs, ultrasonography, and digital rectal examination (DRE) are non-invasive methods of diagnosing BPH. In contrast, fine-needle aspiration (FNA) and biopsy, as the gold standard of diagnosis, are invasive methods. The DRE has low sensitivity because it only examines the caudal part of the prostate. It is not suitable for large or small breeds, so it does not apply to all dogs (9). Clinically, a prostate biopsy is the gold standard, and FNAs are not routinely used to distinguish BPH from other prostatic lesions in living animals (10).

The ultrasound examination gives information about the size, shape, contour, echogenicity, symmetry, and adjacent soft tissues. Prostate size in dogs can be determined by the relative prostatic size (*S*_rel_) (11). The ratio between the prostate volume of an individual dog and the normal prostate volume is computed. As a result, dogs with a *S*_rel_ > 1 had larger prostates than expected (11). In recent studies, circulating biomarkers such as canine prostatic specific esterase (CPSE) have been studied as prostate health markers. It was supported by the serum CPSE level in BPH-affected dogs being higher than that of healthy dogs. There is no data on the concentration of a specific esterase in dogs with different prostate disorders (12–14). CPSE displays promising potential for detecting BPH. However, it is not commonly used in clinical diagnosis as the prostate-specific antigen (PSA) in humans (12).

Platelets mediate hemostasis play a significant role in host inflammation. Hence, in a resting state, platelets circulate in the blood. Several platelet indices that may serve as proxy markers for platelet activation can be determined using optical-based automated hematology analyzers (15, 16). Two factors may contribute to the increased number of large platelets in the bloodstream during inflammation. (1) increased cytokine concentrations result in thrombocytosis, which produces large immature platelets regardless of platelet concentrations; (2) after activation, platelets undergo a change in shape, resulting in the formation of small protrusions on their surface (17).

MPV increases have been linked to acute appendicitis (18), pancreatitis (19), infectious endocarditis (20), myocardial infarction (21) and malaria (22). Several studies have found that increased MPV at admission and during hospitalization predicts a poorer outcome in critically ill patients with sepsis (23). Within 24 h of diagnosis, the only platelet parameter associated with increased mortality in dogs with surgically treated septic peritonitis was MPV (24). The higher MPV indicates that activated platelets are associated with inflammation. In a canine model, MPV and PDW can be used to diagnose and monitor dogs with endotoxemia (25).

Platelets play a substantial role in inflammatory responses, hemostasis, thrombosis, and wound healing by producing and secreting inflammatory and pro-inflammatory cytokines, such as IL-8 and 6 (26). Cytokines, chemokines, and inflammatory mediators are believed to be important in the pathogenesis, symptoms, and progression of BPH (27). Alternatively, thrombosis is affected by androgenic hormones such as dihydrotestosterone and the ratio of androgen to estrogen, affecting platelet indices (28). As a result of the alteration in the androgen-to-estrogen ratio in BPH, changing platelet indices should be expected. As a result, the purpose of this study was to compare platelet indices in dogs with clinical BPH and healthy controls. In addition, to see if there is a link between relative prostatic size (*S*_rel_) and platelet indices in BPH dogs.

## Materials and methods

### Study design

A prospective, case-controlled observational study on dogs with naturally occurring clinical BPH was conducted at the Semnan Veterinary Academic Hospital (SVAH) between August 2018 and July 2019. The current study was approved by the Faculty of Veterinary Medicine's Ethics Committee in biological research (REC12-96). All experiments were carried out in accordance with national guidelines and applicable national laws on animal protection. The study used privately owned dogs of various breeds and enrolled them only with the owner's permission.

### Study population

This study included client-owned intact male dogs over the age of four who were clinically and ultrasonographically diagnosed with BPH and were otherwise healthy. The reproductive history (fertility or previous mating), physical examination, and clinical signs such as tenesmus, dysuria, constipation, stilted gait, hemorrhagic urethral discharge, and intermittent hematuria (7), as well as *S*_rel_ detected by ultrasonography, were used to make a presumptive diagnosis of BPH (29). Platelet clumps visible in the blood film, urinary turbidity, history of renal failure (protein-losing nephropathy, chronic kidney disease, and ureteral obstruction), and coagulation disorders (thrombocytopenia, increased PT, and aPTT), current antiandrogenic and anticoagulant medication, and asymmetric and heterogeneous prostate on ultrasonographic findings were all exclusion criteria.

### Ultrasonographic evaluation

Two independent radiologists evaluated the prostate appearance, border, and size using a 5–7.5 MHz micro convex probe (MyLab™ClassC; Esaote Spa, Genua, Italy) based on a standardized protocol (29). The individual prostatic volume (IPV) and the expected prostatic volume (EPV) were calculated according to Kamolpatana et al. (11) and Sannamwong et al. (29) as IPV = (1/2.6) ^*^ (Length × Width × Height) + 1.8 cm^3^ and EPV = 0.33 × body weight (BW) (kg) + 3.28, respectively. The IPV was compared with the EPV to obtain a relative prostatic size (*S*_rel_ = IPV/EPV). The dogs were divided into two groups based on *S*_rel_ and clinical signs of BPH; dogs with *S*_rel_ > 1 and BPH-related clinical signs (Group A; *n* = 27) and dogs with *S*_rel_ < 1 with no clinical signs of prostate disorders (Group B; *n* = 11) (Supplementary material). The prostate gland was also evaluated by sonography for cysts, parenchymal homogeneity, and shape symmetry.

### Sample collection and laboratory methods

Peripheral blood was collected into plain and EDTA vacutainer tubes prior to any treatment (Deltalab). Within 1 h of blood collection, CBCs and blood smears were performed. Blood smears were prepared and stained with Giemsa (Merck), and platelet aggregation was assessed immediately after blood collection. PLT, PCT, MPV, and PDW were measured using a dog-validated automatic blood cell counter (Nihon Kohden, Celltac Alpha VET MEK-6550, Japan).

### Statistical analysis

Statistical analysis was performed using MedCalc software (MedCalc, version 15.2, Statistical Software). Data was tested for normality using the Kolmogorov-Smirnov test and reported as mean ± standard deviation (SD) when normally distributed and median and interquartile range when not normally distributed. Threshold platelet indices were calculated by the receiver operating characteristic (ROC) analysis for diagnosing BPH. The correlation between *S*_rel_ and platelet indices was determined using Spearman's rank correlation coefficient. Hence, the correlation coefficient (*r*); 0.20–0.39, 0.40–0.59, and 0.60–0.79 are weal, moderate and strong, respectively.

## Results

### Ultrasonographic findings of the canine prostate

**Figure 1:**
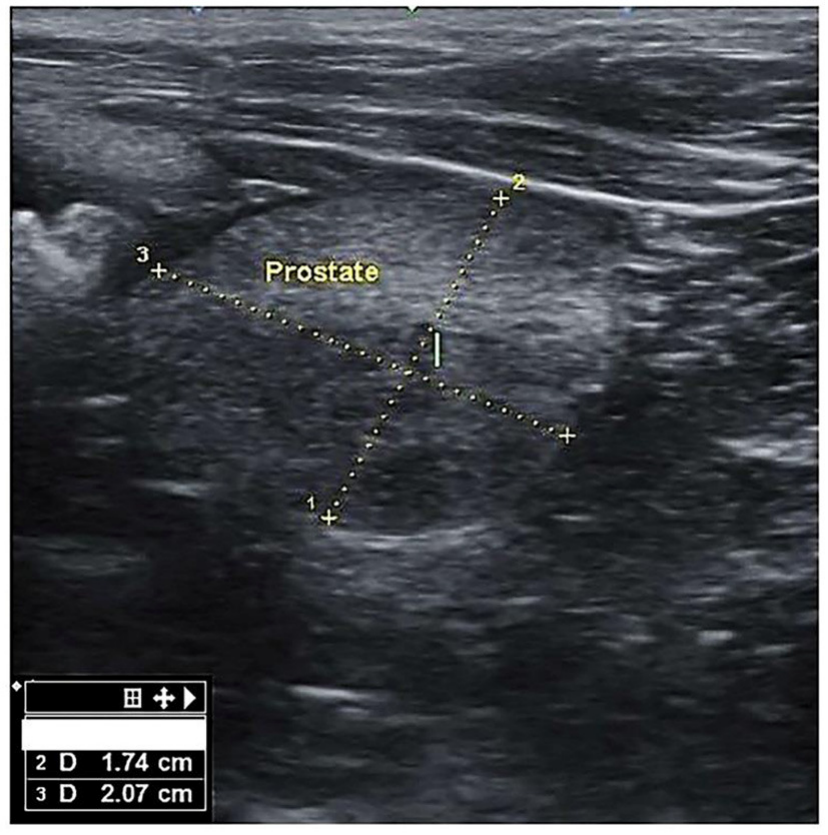
Transverse section of the prostate gland in dogs suffering from BPH, showing the height (1) and width (2).

There mainly were symmetrical enlargements and homogeneous parenchyma in the prostates of dogs in BPH dogs (Figure 1). The prostate of some cases (*n* = 3) was asymmetric and heterogeneous because of intraparenchymal cysts and nodules in the ultrasound evaluation, and these cases were excluded from the study. There were no visible changes in the prostate parenchyma in the B group. Group A included six mongrels and 18 other dogs from six defined breeds, i.e., Terrier (*n* = 10), Siberian Husky (*n* = 3), Spitz (*n* = 2), German Sphered (*n* = 1), Belgian Malinois (n = 1), and Pekingese (*n* = 1). The weight ranged from 3 to 55 kg, with a median of 8.30 kg (interquartile range [IQR]: 6.1–16). Age ranged from 4 to 15 years, with a median of 6.0 years (IQR: 5.25–7.75). Group B included three mongrels and eight other dogs from five defined breeds, i.e., Shih tzo (*n* = 3), Terrier (*n* = 2), German Sphered (*n* = 1), Pekingese (*n* = 1), and Pomer (*n* = 1). Weight ranged from 4.5 to 30 kg, with a median of 7.0 kg (IQR: 4.5–30.0). Age ranged from 4.5 to 10 years, with a median of 5.50 years (IQR: 5.0–7.5).

### Comparison of *S*_rel_ and platelet indices between groups

**Figure 2:**
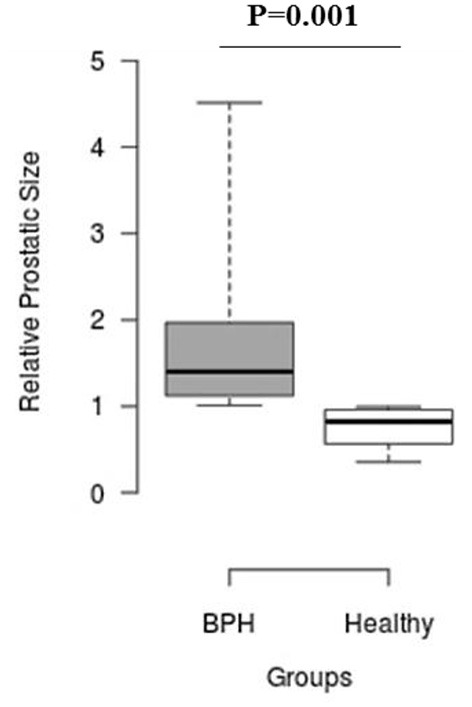
Box plots of relative prostatic size (*S*_rel_) in BPH and Healthy groups. Center lines show the medians; box limits indicate the 25th and 75th percentiles. *n* = 24, 11 sample points.

**Figure 3:**
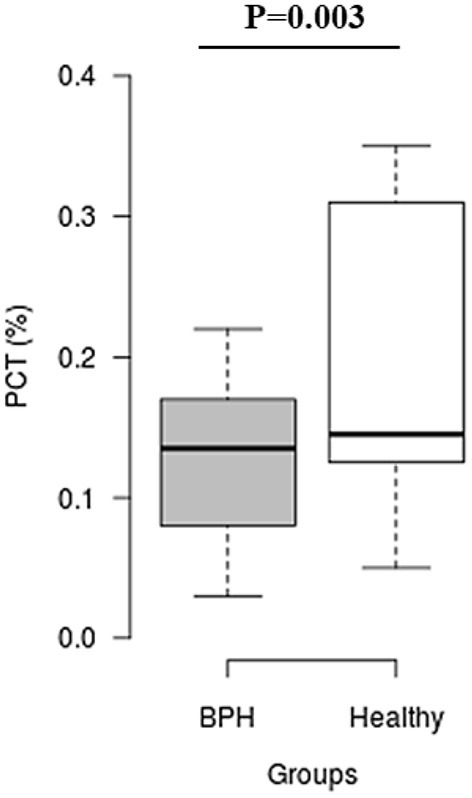
Box plots of plateletcrit (PCT) in BPH and Healthy groups. Center lines show the medians; box limits indicate the 25th and 75th percentiles. *n* = 24, 11 sample points.

**Figure 4:**
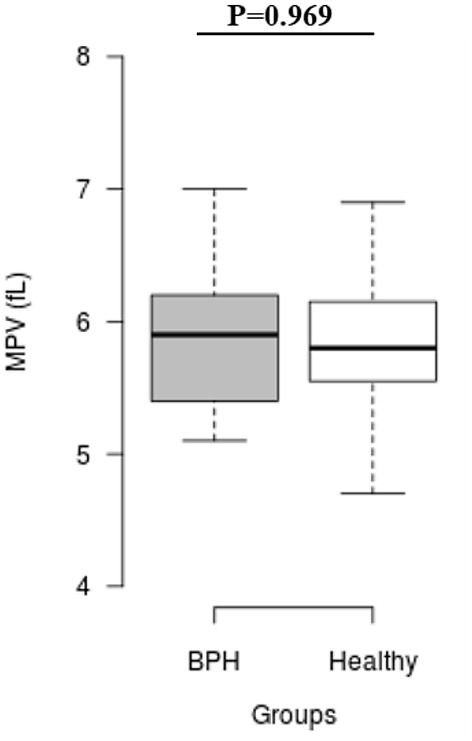
Box plots of mean platelet volume (MPV) in BPH and Healthy groups. Center lines show the medians; box limits indicate the 25th and 75th percentiles. *n* = 24, 11 sample points.

**Figure 5:**
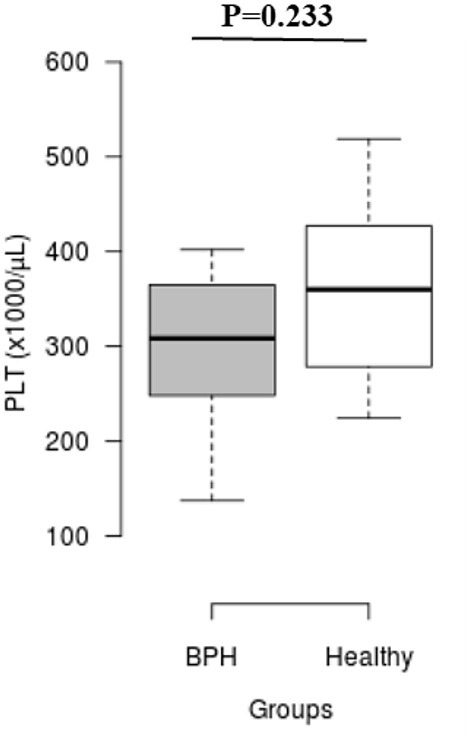
Box plots of platelet counts (PLT) in BPH and Healthy groups. Center lines show the medians; box limits indicate the 25th and 75th percentiles. *n* = 24, 11 sample points.

**Figure 6:**
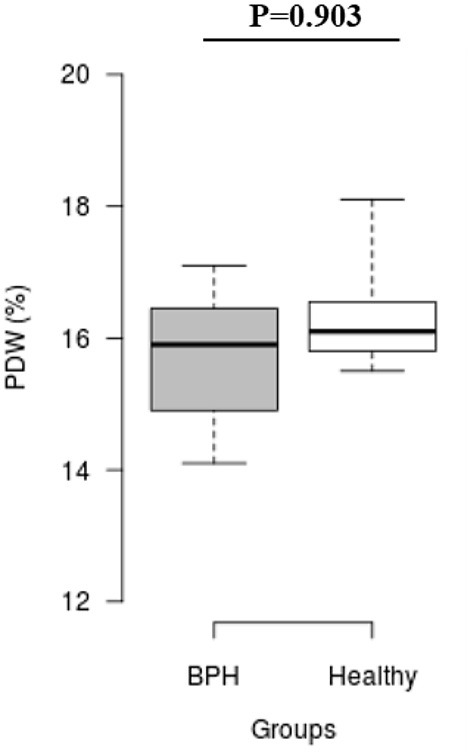
Box plots of platelet distribution width (PDW) in BPH and Healthy groups Center lines show the medians; box limits indicate the 25th and 75th percentiles. *n* = 24, 11 sample points.

The median *S*_rel_ in group A was significantly higher than in group B (1.40; IQR: 1.12–1.96 vs. 0.82; IQR: 0.55–0.96, *P* = 0.001) (Figure 2). Most of the platelets of group B in blood smears were uniform and medium in size, whereas platelets of all dogs from group A showed anisocytosis. Platelet clumping was not detected in any of the blood smears. The mean PCT of dogs in group A decreased significantly (*P* = 0.003) compared with those in group B (13.54 ± 1.19%) and (0.20 ± 0.11%) (Figure 3). Compared to group B, a higher MPV (*P* = 0.969; Figure 4) and a lower mean of PLT (*P* = 0.233; Figure 5) and PDW **(***P* = 0.903; Figure 6) were noted in group A.

### Correlation between *S*_rel_ and platelet indices

A significant negative correlation was found between *S*_rel_ and PLT and PCT (*r* = −0.388; *P* = 0.02 and *r* = −0.402; *P* = 0.01, respectively). The correlation coefficient (Table 1) shows that MPV and PDW were weakly correlated (*r* = 0.214; *P* = 0.21 and *r* = 0.036; *P* = 0.83, respectively).

**Table 1:** Correlation between relative prostatic size and platelet indices.

**Variables**	**Correlation coefficient (R)**	***P*** **-value**
PLT (× 10^3^/μL)	−0.388	0.02
PCT (%)	−0.402	0.01
MPV (fL)	0.214	0.21
PDW (%)	0.036	0.83

PLT, platelet counts; PCT, plateletcrit; MPV, mean platelet volume; PDW, platelet distribution width.

### Cut-off values of platelet indices based on *S*_rel_ for diagnosing clinical BPH

**Figure 7:**
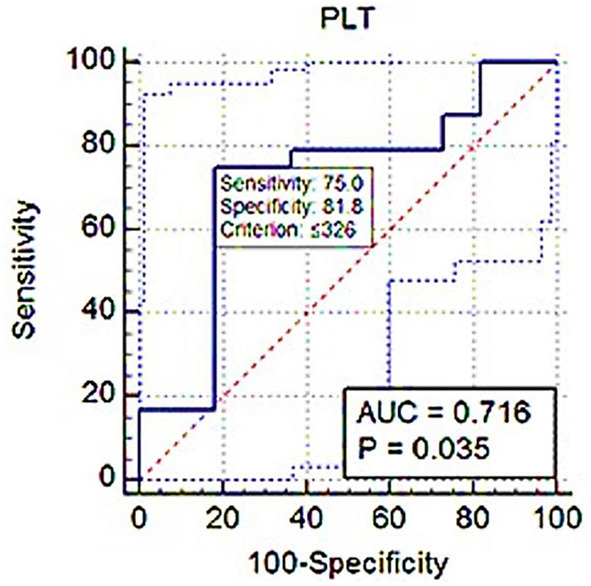
ROC (receiver operating characteristic) curve analysis of PLT for *S*_rel_ > 1. The solid blue line between the two blue dashed lines is the corresponding cut-off point with sensitivity and specificity. The blue dashed lines, one on the upper left side and the other on the lower right side of the solid blue line, are the upper and lower 95% confidence bounds, respectively.

**Figure 8:**
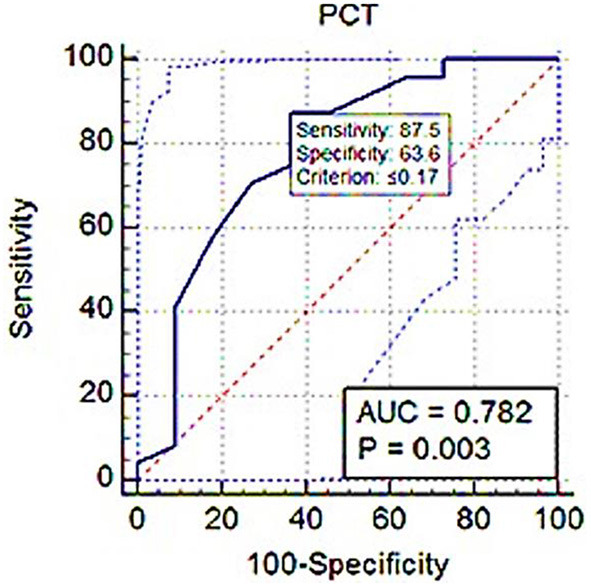
ROC (receiver operating characteristic) curve analysis of PCT for *S*_rel_ > 1. The solid blue line between the two blue dashed lines is the corresponding cut-off point with sensitivity and specificity. The blue dashed lines, one on the upper left side and the other on the lower right side of the solid blue line, are the upper and lower 95% confidence bounds, respectively.

ROC analysis showed PLT threshold for estimating *S*_rel_ > 1 with 75% sensitivity, 71.82% specificity (AUC; 0.716) was ≤ 326 × 10^3^ (Figure 7). The PCT-based cut-off value *S*_rel_ > 1 was ≤ 0.17% (AUC; 0.782, 87.5% sensitivity, 63.64% specificity; Figure 8).

## Discussion

The most common prostate disorder in dogs is BPH, which is followed by hormonal atrophy, squamous metaplasia, cysts, neoplasia, and inflammation (24). This study looked at the relationship between platelet indices and *S*_rel_ in BPH dogs, taking into account evidence linking these indices to a variety of non-hematological illnesses in dogs (30, 31). Platelet mass is measured with the PCT and calculated using PLT and MPV (32). The studies evaluating PCT in malignant and benign tumors were limited. A higher PCT was found in benign epithelial ovarian masses than in healthy individuals, but the difference was insignificant (33). In this study, PCT was significantly lower in BPH dogs compared to healthy ones (*P* = 0.003). A reduction in platelet production by the bone marrow or an increase in giant platelet consumption by pro-inflammatory molecules may be associated with changes in these indices (34, 35). It has been shown that blood vessels protect themselves from tumor cells by natural killer cells, and tumor cells are protected by platelets from natural killer cells, resulting in lower platelet consumption in the early stages of tumors (36, 37). Platelets play a role in tumor angiogenesis and inflammation by producing and releasing vascular endothelial growth factor (VEGF) (38).

In terms of platelet function, large platelets are more active than small platelets (39). MPV has been studied in a variety of cancer types (40). A study showed that patients with benign goiter had significantly higher MPV levels than healthy people (41). Rifaioglu et al. (42) reported that asymptomatic prostatitis patients had a higher MPV than healthy individuals (34). However, other studies have presented conflicting results regarding the influence of prostate disorders on platelet indices (42–47). In the present study, a significant difference was not observed in MPV between the two groups.

PDW is a morphometric indicator used to describe the distribution of peripheral platelet size. There have been few studies on the relationship between various tumors and PDW, and even those that have been conducted have yielded contradictory results. PDW was significantly higher in ovarian cancer patients than in healthy people (48). However, some studies found a significant decrease in PDW levels in patients with non-small cell lung cancer, breast cancer, and malignant adnexal cancer (49–51). This study found no significant difference in PDW levels between BPH and healthy dogs.

Local hypoxia incidence in BPH exacerbates inflammation since low levels of reactive oxygen species encourage neovascularization and angiogenesis. Prostate stromal cells can also increase VEGF-2, VEGF-7, and tumor growth factor-β (TGF-β) secretion, all of which play important roles in determining prostatic growth rates in response to hypoxia (52, 53). The PLT and PCT correlate negatively with the relative prostatic size in BPH dogs. A reduction in platelet production by the bone marrow or an increase in giant platelet consumption by proinflammatory molecules may be associated with changes in these indices (34, 35). In inflammatory conditions, the relationship between PLT and MPV is inverse. As thrombopoiesis accelerates, large active circulating platelets migrate to inflamed sites where they are intensely consumed (51).

Eventually, in the ROC curve analysis, the AUC value was from 0.5 to 1; the closer the AUC value was to 1, the better the diagnostic effect. An AUC of between 0.7 and 0.9 showed high accuracy. In this study, the PLT and PCT use to distinguish BPH dogs from healthy dogs had a large AUC, increased sensitivity, and high precision.

Findings from the study by Fu et al. (44) suggest that MPV and PDW are associated with prostate cancer in patients. A specific mechanism showing a relationship between prostate cancer and MPV and PDW has not been precisely defined.

The function of platelets may differ based on sex hormones. Researchers suggest sex hormones have non-genomic effects on platelets or genomic impacts on megakaryocytes. Thromboxane A2 receptors were increased in healthy men by testosterone (*T*) treatment, which caused platelet activation. The arachidonate receptors increasing on blood platelets have proposed the link between *T* and platelet reactivity (54). However, *T*-stimulated endothelium also secretes nitric oxide (NO) that indirectly inhibits platelet activation (55). Platelet variable values are affected by thrombopoiesis intensity. Thrombopoiesis is affected by androgen hormones and the androgen-to-estrogen ratio (38). Based on Karolszak et al.'s (45) findings, androgen concentrations negatively correlate with various measurements of platelet morphology, such as MPV, PCT, PDW, and platelet large cell ratio (P-LCR). Therefore, changes in platelet indices should be expected because of the alteration in the androgen concentrations and androgen to estrogen ratio in BPH.

The current study discovered that: (1) PLT, MPV, and PDW measurements in dogs with BPH were not significantly different from controls; (2) PCT values in BPH dogs were significantly lower; (3) PLT and PCT were moderately negatively correlated with *S*_rel_; and (4) PLT and PCT sensitivity and specificity calculated by ROC analysis for estimating *S*_rel_ can be used in conjunction with other diagnostic markers to evaluate clinical BPH in dogs. The findings of this study support the use of platelet indices like PLT and PCT to detect clinical BPH in dogs. However, more research is needed to confirm their utility in conjunction with other previously described diagnostic factors.

The current study has a limitation due to a lack of access to a valid and specific CPSE-ELISA kit. As a result, we were unable to determine whether the relationship between platelet indices and this diagnostic marker differed between BPH dogs and healthy controls. More research is needed to determine whether there are any statistically significant differences.

## Data availability statement

The raw data supporting the conclusions of this article will be made available by the authors, without undue reservation.

## Ethics statement

The animal study was reviewed and approved by Iranian Society for the Prevention of Cruelty to Animals and Semnan University Research Council supervised this study under Iranian animal Ethics guidelines. Written informed consent was obtained from the owners for the participation of their animals in this study.

## Author contributions

HH: study design, data, and primary author of the manuscript. MA-h: study design, data, result analysis, and manuscript editing. MM: study design and manuscript editing. DS: data and result analysis. RN: manuscript editing. All authors contributed to the article and approved the submitted version.
