# Supplementary material for: Assessment of the utility of platelet indices to diagnose clinical benign prostatic hyperplasia in dogs
Source: Front Vet Sci. 2022 Dec 8;9:1031292. doi: 10.3389/fvets.2022.1031292 (PMC9772470; doi:10.3389/fvets.2022.1031292)
Supplement: Supplementary file 1 [file Table_1.DOCX]

**TABLE**  Age, body weight, breed, individual prostatic volume, expected prostatic volume and relative prostatic size, clinical signs and groups in the 35 adult male dogs

| Dog | Age (years) | BW (kg) | Breed | IPV | EPV | S_rel_ | Clinical signs | Group | |
| --- | --- | --- | --- | --- | --- | --- | --- | --- | --- |
| 1 | 5.5 | 12 | husky | 7.404412308 | 7.24 | 1.022709 | + | | A |
| 2 | 10 | 5.5 | terrier | 5.313461538 | 5.095 | 1.042878 | + | A | |
| 3 | 5.2 | 3 | mix | 4.87435792 | 4.270 | 1.141536 | + | A | |
| 4 | 6.2 | 55 | german | 42.30276923 | 21.43 | 1.973998 | + | A | |
| 5 | 7 | 6.1 | terrier | 6.740061538 | 5.293 | 1.273392 | + | A | |
| 6 | 13 | 5.6 | terrier | 5.484546923 | 5.128 | 1.069529 | + | A | |
| 7 | 7 | 5.3 | terrier | 7.098076923 | 5.029 | 1.411429 | + | A | |
| 8 | 4.9 | 12 | terrier | 8.26153846 | 7.240 | 1.141096 | + | A | |
| 9 | 6 | 26 | husky | 53.55515462 | 11.86 | 4.515612 | + | A | |
| 10 | 5 | 7.8 | mix | 7.583347692 | 5.854 | 1.295413 | + | A | |
| 11 | 8 | 27 | mix | 28.72315385 | 12.19 | 2.356288 | + | A | |
| 12 | 4.1 | 30 | mix | 24.95384615 | 13.18 | 1.893312 | + | A | |
| 13 | 5.5 | 5.8 | spitz | 5.262384615 | 5.194 | 1.013166 | + | A | |
| 14 | 6 | 28 | mix | 32.04708923 | 12.52 | 2.559672 | + | A | |
| 15 | 15 | 8 | terrier | 6.229163077 | 5.92 | 1.052223 | + | A | |
| 16 | 7.9 | 18 | malinios | 18.84820154 | 9.22 | 2.044273 | + | A | |
| 17 | 14 | 12 | mix | 11.20004615 | 7.240 | 1.547025 | + | A | |
| 18 | 6 | 8.2 | spitz | 11.73138462 | 5.986 | 1.959804 | + | A | |
| 19 | 5.6 | 9 | terrier | 6.931487308 | 6.25 | 1.109038 | + | A | |
| 20 | 5.1 | 6.1 | terrier | 14.78414154 | 5.293 | 2.79315 | + | A | |
| 21 | 7.5 | 8.4 | terrier | 8.418296923 | 6.052 | 1.390994 | + | A | |
| 22 | 5.3 | 7.8 | terrier | 7.055252308 | 5.854 | 1.205202 | + | A | |
| 23 | 6 | 14 | husky | 11.20361538 | 7.9 | 1.418179 | + | A | |
| 24 | 7.7 | 6.7 | pekingese | 7.767530769 | 5.491 | 1.414593 | + | A | |
| 25 | 7.5 | 6 | terrier | 3.068723077 | 5.26 | 0.583407429 | - | B | |
| 26 | 10 | 25 | mix | 11.41666462 | 11.53 | 0.990170392 | - | B | |
| 27 | 5.2 | 4.5 | shihtzo | 4.529336923 | 4.765 | 0.950542901 | - | B | |
| 28 | 7 | 7 | pekingese | 4.292936538 | 5.59 | 0.76796718 | - | B | |
| 29 | 5 | 30 | mix | 13.10769231 | 13.18 | 0.994513832 | - | B | |
| 30 | 4.5 | 4.5 | pomer | 2.612508462 | 4.765 | 0.548270401 | - | B | |
| 31 | 5 | 30 | mix | 4.676185385 | 13.18 | 0.354794035 | - | B | |
| 32 | 5.5 | 30 | german | 5.727115769 | 13.18 | 0.434530787 | - | B | |
| 33 | 6 | 7.5 | terrier | 4.737692308 | 5.755 | 0.823230636 | - | B | |
| 34 | 7.5 | 7 | shihtzo | 5.395384615 | 5.59 | 0.965185083 | - | B | |
| 35 | 4.8 | 4.5 | shihtzo | 4.025192308 | 4.765 | 0.844741303 | - | B | |

BW, body weight; IPV, individual prostatic volume; EPV, expected prostatic volume; S_rel_, relative size.
